# What SARS-CoV-2 Variants Have Taught Us: Evolutionary Challenges of RNA Viruses

**DOI:** 10.3390/v16010139

**Published:** 2024-01-18

**Authors:** Shymaa E. Bilasy, Tutik Sri Wahyuni, Mohamed Ibrahim, Ahmed El-Shamy

**Affiliations:** 1 College of Dental Medicine, California Northstate University, Elk Grove, CA 95757, USA; shymaa.bilasy@cnsu.edu; 2 Department of Pharmaceutical Science, Faculty of Pharmacy, Natural Product Medicine Research and Development, Institute Tropical Disease, Airlangga University, Surabaya 60286, Indonesia; tutik-s-w@ff.unair.ac.id; 3 College of Biotechnology, Misr University for Science and Technology, Giza, Egypt; mohamed.ibrahim@must.edu.eg; 4 Master of Pharmaceutical Sciences Program, College of Graduate Studies, California Northstate University, Elk Grove, CA 95757, USA

Since its discovery in 2019, SARS-CoV-2 still makes the headline news. SAR-CoV-2 is an emerging RNA virus that spread from a seafood market in Wuhan, China, and caused the COVID-19 pandemic. SARS-CoV-2 is a positive sense single-stranded RNA virus of about 30 kb in length that is derived from the *Coronaviridae*. Up to September 2023, more than 770 million COVID-19 cases and 6.9 million deaths were reported to the World Health Organization (WHO). Around 37 variants of SARS-CoV-2 were reported and they were categorized as variants of concern (VOC) or variants of interest (VOI) or variants being monitored (VBM) [1]. The WHO labeled SARS-CoV-2 Omicron (B.1.1.529) as a variant of concern in November 2021 [2]. This variant was first reported in South Africa, but it quickly became the dominant circulating SARS-CoV-2 worldwide [3,4]. The Omicron viruses continued to genetically evolve with more sub-lineages added to its phylogenetic tree. The genetic divergence of Omicron has been associated with changes in the viral transmissibility, virulence, and ability to evade protective immune response conferred by natural infection or vaccination [5].

To shed light on the evolutionary behavior of RNA viruses and how it shapes their epidemiolocal fitness and pathological features, *Viruses* developed a Special Issue entitled “What SARS-CoV-2 Variants Have Taught Us: Evolutionary Challenges of RNA Viruses”. This issue included twenty-four research topics comprising different aspects of SARS-CoV-2 infection. Viral lineages in different geographical locations were discussed by several authors. Omicron and its sub-variants were responsible for COVID-19 infections in several countries. Given the large number of mutations in Omicron compared to its previous predecessors, the existence of missing SARS-CoV-2 variants was addressed in the *Viruses* Special Issue. Interestingly, phylogenetic analyses suggested the presence of intermediate variants between SARS-CoV-2 Omicron and Delta variants, which might have not been documented. In addition, developing rapid and accurate diagnostic methodology was another point explored in this Special Issue. Targeted reverse-transcriptase quantitative polymerase chain reaction (RT-qPCR) was highlighted in this Special Issue to accurately identify new variants. Compared to NGS, the targeted RT-qPCR-based method is more cost-effective and flexible and can provide near real-time changes in variant prevalence.

In general, RNA viruses have high genetic variability due to fast, low-fidelity replication. Mutations, recombination, and reassortment are the main mechanisms responsible for genetic change and evolution [6]. Those changes can eventually affect viral fitness. In this *Viruses* Special Issue, the genome architecture of different SARS-CoV-2 variants was analyzed with the aim of discovering mutations correlated with viral pathogenicity. A higher number of mutations were associated with mortality cases in both Delta and the Omicron variants. Further, compared to MERS-CoV and SARS-CoV-1, the SARS-CoV-2 genome was biased towards a lower GC dinucleotide content, which may explain its moderate virulence.

Several articles addressed the immune response to SARS-CoV-2 infection, which revealed several immunogenic regions. Immunogenic regions may be correlated to the disease severity and/or potentially used as serological markers.

Numerous mutations in Omicron and its sub-lineages altered the transmission dynamics and pathophysiology. Sun et al. reported neutralization assays using diluted plasma samples from COVID-19 convalescent patients using a SARS-CoV-2 pseudovirus. This model contained the SARS-CoV-2 spike protein on an HIV-1 backbone and a luciferase reporter gene [7]. Although pseudoviruses cannot reflect the behavior of the full virus, they are considered a versatile research tool that can be conveniently performed at a lower biosafety level and can easily introduce mutations to reflect the newly discovered variants. Among the Omicron viruses, several variants and subvariants are classified as “variants being monitored” according to the updated guidelines developed by the WHO [1]. For some time, the BA.2.86 and XBB.1.1, a recombinant form of two BA.2 sub-lineages, were dominating the community spread. Recently the EG.5 (Eris) accounts for 25% of the new cases. The EG.5 variant descends from the XBB.1.9.2 subvariant. However, it contains an additional FLip mutation, Phe456Leu, and its subvariant, EG.5.1, has another spike protein mutation (Q52H) [8]. Therefore, pseudoviruses can be instrumental in exploring the significance of those mutations. 

In conclusion, as an emerging RNA virus, SARS-CoV-2 will continue to evolve, and the threat of a viral outbreak will always be present. This virus has displayed a remarkable ability to mutate and generate new variants, and it has been proven to be a highly adaptable and evolving pathogen, leading to public health concerns. This Special Issue of *Viruses* sheds light on the evolutionary nature of SARS-CoV-2 and its potential implications for public health. Recognizing the ever-evolving nature of SARS-CoV-2 underscores the importance of continued surveillance, vaccination strategies, and adaptable public health responses to address this dynamic threat.
